# Oinomania; or, the Mental Pathology of Intemperance

**Published:** 1855-04-01

**Authors:** 


THE JOURNAL
OF
PSYCHOLOGICAL MEDICINE
AND
MENTAL PATHOLOGY.

APRIL 1, 1855.

### Art. I.—OINOMANIA; OR, THE MENTAL PATHOLOGY OF INTEMPERANCE.

In our last Number we briefly referred to a noble effort now in progress in the United States of America to establish an "Inebriate Institution" for the reception and treatment of that form of insanity, or monomania manifesting itself principally in a morbidly uncontrollable propensity for alcoholic stimulants. We do not refer to drunkenness or intemperance in the popular signification of the terms, but to a *phase* of *disordered mind*, of *cerebral* disease, the prominent symptom being an irresistible yearning for intoxicating drinks. The experienced physician and pathologist may easily distinguish between ordinary habits of intemperance and "fits" of drunkenness and the form of insanity to which we allude. This mental disorder exists to a frightful extent in all classes of society, from the highest to the lowest grade, and is destructive, beyond all conception, to domestic happiness, to national prosperity, and to social and private morals. It is a form of alienation of mind which has, to a great degree, escaped medical and general observation, and may therefore be classed among the unrecognised phases of mental derangement. The subject, we admit, is a delicate and difficult one to grapple with, mainly in consequence of the prevailing fallacies existing in the public mind with respect to morbid affections of the intellect. This must not, however, deter us from breaking ground upon so important a matter.

There have been large contributions to the literature of drunkenness in England of late. Twenty years ago a numerous Committee of the House of Commons collected a vast mass of information on the subject by special inquiry.* Dr. Carpenter has recently published a

* "Report of an Inquiry into Drunkenness," ordered by the House of Commons to be printed, 5 August, 1834.

valuable essay on the subject,* and we have just received an eloquent and well-written work on the Pathology of Drunkenness, from the pen of an intelligent Scotch physician.† To these, other names of English authors might be added. Very able writers in Germany, Sweden, Denmark, &c., have also added largely to our knowledge. Amongst these may be mentioned Friedreich, Brühl-Cramer, Barkhausen, Most, Heinroth, Lippich, Rösch, Huss, &c. Twenty years of uninterrupted inquiry, and an experience of the affection most widely extended, must be considered sufficient for a thorough elucidation of its general pathology and therapeutics.

*Polydipsia ebriosa*, or drinking to drunkenness, shows itself in various ways. First in order comes the habitual sot—the man or woman who drinks from early morn to dewy eve, and who is either always drunken, or more deeply drunken, either at night or twice in each day. This, for practical purposes, may be termed *continuous* drunkenness. Next is the *intermittent* form, in which there are two or three days of continuous drunkenness, then two or three days of entire or partial abstinence from alcoholic drinks. This is seen most typically in mechanics of drunken habits, who drink from Saturday night to Monday night or Tuesday morning, and then, going to their usual employments, continue sober for the rest of the week. This intermittent drunkenness is, for the most part, unconnected with any morbid changes in the nervous system; and in this essential respect it differs from continuous drunkenness, and from an intermittent form, which recurring at much longer intervals may be termed *paroxysmal* drunkenness. Writers mention other forms, but they do not present points of difference sufficiently important for recognition as distinct from the preceding, being, in fact, those forms in varying degrees of intensity, or commingled, or else instances complicated with other diseases of the nervous system.

*Polydipsia ebriosa*, we hardly need say, is not necessarily insanity, for the "thirsty soul" may usually be rendered amenable to those motives which regulate the conduct of rational or sane men. That drunkenness can only be designated insane (*Polydipsia ebriosa insana*, *Dipsomania*, *Oinomania*) which is induced by the *action of an impulsive desire for stimulant drinks, uncontrollable by any motives that can be addressed to the understanding or conscience: in which self-interest, self-esteem, friendship, love, religion, are appealed to in vain; in which the passion for stimulant drinks is the master-passion, and subdues to*

* "On the Use and Abuse of Alcoholic Liquors in Health and Disease." Prize Essay. By William B. Carpenter, M.D., F.R.S., &c. 1850.

† "The Pathology of Drunkenness: a View of the Operation of Ardent Spirits in the Production of Disease." By Charles Wilson, M.D. 1855.

*itself every other desire and faculty of the soul.* We say stimulant *drinks,* for we speak of intoxication in the ordinary sense of the word; but, in truth, the mania has a wider range of victims than the drunkard, and includes all those who indulge uncontrollably in narcotic and stimulating drugs, as the drunkard indulges in alcoholic drinks. The confirmed *opium-eater* is, therefore, virtually, although not etymologically, in the same category as the oinomaniac; for he is equally the slave to his insane appetite. A word is yet to be invented which shall designate correctly the form of mental aberration to which we refer. We will not venture the attempt, but will simply define it to be an insane appetite for those agents which received into the blood develop the pleasurable feelings of mental or corporeal well-being—a state of contentment, ease of mind or of body, attributed, in popular language, to "the spirits"—so as to *raise* "the spirits," and induce satisfaction, happiness, and cheerfulness, or else antagonize the opposite condition to this feeling of mental or physical well-being, and so counteract unhappiness, "lowness of spirits," or depression of the vital and mental powers.

The circumstances under which nervine stimulants are taken are very various and widely different. The most general excitant of the appetite is that condition of the mind in which there is simply a desire for pleasureable excitement and little power of will to resist the temptation to gratify the desire. Persons of this class are numerous in the world. They have hereditarily a large capacity for physical enjoyment, conjoined with feeble intellect or judgment, or, if not feeble, are highly susceptible to all painful and pleasurable impressions, and *are often the offspring of persons who have indulged in stimulants, or who have weakened the cerebral organization by vicious habits and undue mental labour*. Not unfrequently there is a hereditary tendency in the family to hysteria, convulsive affections, eccentricity of character, oinomania, suicide; perhaps, in one branch or member, great talent; in another, a weakness of mind amounting almost to imbecility. The immediate result of the action of alcohol upon the system of a person in health is a more vigorous and agreeable discharge of all the vital functions. The blood courses more rapidly through the bloodvessels; the voice is more sonorous; the eye more bright; the muscular system braced up. But it is the nervous system which responds most readily to the stimulus. The intellect is clearer, the imagination more vivid, the memory more distinct, the thoughts more definite; in short, all the faculties are exalted. As to the emotions—joy, exhilaration, and good-fellowship are the principal results: as to the appetites, an increased development. If the stimulus be taken in larger quantity so as to act as a poison—*i.e.*, to *derange* the functions—morbid phenomena in connexion with the encephalic centres are induced. The individual, perhaps, manifests more clearly his natural infirmities of character. The irritable and ill-tempered become quarrelsome; the silly, good-natured, and the foolish are officiously urgent in offers of kindness, laugh wildly and are tickled by trifles; and the melancholic become maudlin sentimental. Sometimes an individual passes through successive phases of mental change—as thus: at the first half-bottle of wine he is energetic, dignified, and decided, and his conversation is of affairs of moment. At the second he is mirthful, and indulges in the song and the jest. His motto is "*dulce est desipere in loco.*" At the third he is emotional and sentimental, easily moved to tears, and becomes, perhaps, amorous, or religious, as individual circumstances may determine. At the fourth, he is quarrelsome and incoherent; at the fifth, the cerebellum is manifestly more deeply involved, for he has now lost the power of co-ordinating the muscular system, so that he totters and staggers, or else is affected with *motus vertiginosi*—reeling movements—of various kinds. At the sixth, total abolition of consciousness supervenes, and that condition is attained in which the sot is said to be "*dead*-drunk."

Now, strange as it may appear, this last is the condition desired by many drunkards; it is the summum bonum of their mental and physical existence. Smollett tells us that, in 1742, when distilled liquors were very cheap (being free from duty), the retail dealers put up boards inviting people to be drunk for the small charge of one penny, and dead-drunk for two-pence, with straw free to lie on. Cellars and places strewed with straw were actually provided for these devotees of Lethe. It is to this grovelling felicity that the habitual drunkard usually comes at last, however intellectual, witty, and gay he may have been, when he first began his career.

It is obvious, however, that the stimulant will be taken in quantities and at times in accordance with the corporeal and mental characteristics of the individual. With the educated, wine is drunk to make glad the heart and to invigorate the social feelings. To these results the lyrical poetry of the wine-cup invariably points. Anacreon expressly repudiates the corporeal delights and bestial excesses of the savage; he mingled water, like the ancient Greeks in general, with his wine.

> "No! banish from our board to-night
> The revelries of rude delight;
> To Scythians leave these wild excesses,
> Ours be the joy that soothes and blesses!
> And while the temperate bowl we wreathe,
> In concert let our voices breathe,
> Beguiling every hour along
> With harmony of soul and song."

To antagonize depressing passions, as well as to exhilarate, is often the end of conviviality. Care and anxiety, grief and sorrow, depress the action of the heart, and if they do not make it literally ache, they are often accompanied by a tendency to sigh and a feeling of sinking in the epigastric region. The stimulus of wine has been from time immemorial appreciated as the most ready and effectual antidote to these depressing emotions and sensations.

> "Behold! my boys a goblet bear,
> Whose sparkling foam lights up the air;
> Where are now the tear, the sigh?
> To the winds they fly, they fly!
> Grasp the bowl; in nectar sinking,
> Man of sorrow, drown thy thinking!"

A great proportion of persons who take stimulant agents suffer from an indescribably painful feeling of languor and corporeal illness, which a stimulus is found to remove. This feeling is intolerable to those who, like the English opium-eater, "hanker too much after a state of happiness," or who "cannot face misery with an eye of sufficient firmness," so that the desire to relieve it becomes uncontrollable. Amongst the causes of these sensations may be mentioned those nervous affections which more particularly have their seat in that part of the nervous system which is the seat of the feeling of well-being, and which ministering to the functions of viscera in important relation to life, involve, therefore, the instincts for life and well-being. Certain diseases of the heart, impeding its functional activity or rendering its action painful, induce this depressed condition. Morbid states of the stomach and bowels, either inflammatory or irritative, in which digestion is accompanied by pain and a distressing sensation of weakness in the epigastrium, are amongst the most frequent causes of habitual drunkenness and opium-eating. Of these, chronic gastritis or enteritis are perhaps the most common. To some such state the English opium-eater refers when he asserts that when he first took opium, it was to mitigate a most painful affection of the stomach that occurred under unfavourable circumstances, from depression of spirits, and yielded to no other remedies. The same writer observes that the Dean of ——— and a late under-secretary of state, both used the same words to describe the sensation which induced them first to become opium-eaters,—viz., "that he felt as though rats were gnawing and abrading the coats of the stomach." Hepatic or splenic derangement constitutes another not unfrequent and well-known source of this intolerable *mal-aise*. Equally frequent, but less recognised, is a chronic inflammation or irritation of the mucous membrane of the large intestine, resembling that which attacks the stomach, and, like that, deeply influencing the consciousness as to pleasure and pain. Morbid states of the blood are also amongst the causes of the physical depression, especially those induced by an imperfect supply of oxygen (as when a vitiated atmosphere is habitually breathed), by an imperfect supply of proper nutrient materials, or by the retention of excreta, as the biliary, urinary, &c. Or the morbid condition, on which the distressing feelings depend, may be a change primarily in those ganglionic centres we have referred to, embedded in the cerebral mass, and are certainly in relation with the whole *sympathetic system* of nerves, however imperfectly their anatomy may be known to the anatomist and physiologist. Such a morbid condition may be classed with the true neuralgiæ or nerve-aches, inasmuch as the causes which induce the latter are continually the causes of the former. Perhaps the most common of all is the excessive use or stimulation of any portion of the nervous system.

It is a law of the organism that after a period of action there shall be a period of rest,—after excitement, comes repose. If the latter fail to be induced, pain and morbid action result. The excitement and increased action induced by vinous or narcotic stimulants, is not an exception to the law. On the contrary, the need for repose is strongly expressed by the organism in the general feeling of languor and depression which succeeds to the excitement, so soon as the stimulant effects of the agent have passed off. This feeling does not, however, precede the tranquil rest and refreshing slumber that commonly follow upon labour honestly and temperately pursued; nor is the rest sweet, as in the latter case Nature provides it shall be.

The depression which is felt treacherously points the sufferer to the cause as the remedy for the feeling, and since it effectually answers the purpose—at least temporarily—the thoughtless and imprudent do not hesitate to take it. They have now, indeed, a double inducement to drink,—firstly, to dispel "the blues;" secondly, to secure pleasurable excitement. In this way a poison is taken from day to day, and the man becomes at last the subject of the *maniacal* vice of continuous drunkenness. He has, finally, induced cerebral disease, from the consequences of which he can hardly escape with all appliances and means in his favour that art can afford.

Let us now trace the *pathological* influence of alcohol on the brain, from its first beginnings to the fatal close in mania, general paralysis, or death.

We have already cursorily described the ordinary phenomenon of a fit of drunkenness. The principal characteristic is, that the power of the will over the current of thought and over the actions is weakened, and weakened for the most part, *pari passu*, with the amount of stimulant taken. Now, as this is the principal characteristic of mania, it may be stated that during a fit of drunkenness, the individual is in a condition *quasi* his cerebrum, analogous to that of the insane person. The action of alcohol is therefore concentrated on the brain. As to the different parts of the brain implicated and disordered, it would appear that they are affected from above downwards. Firstly, the cerebral hemispheres, as the seat of intellect and imagination, manifest the action of the poison, next the emotional centres are excited, then the more animal passions are roused, the motor and sensorial centres are next disordered, and finally the sympathetic system. Dr. Carpenter thinks that the specific exciting effect of alcohol upon the nervous centres can only be accounted for by the theory of some special relation between it and nervous matter. And this idea is fully borne out, he thinks, by the results of the experimental researches instituted by Dr. Percy, who found alcohol in the substance of the brains of dogs poisoned by it, in a proportion considerably greater than in an equivalent quantity of blood. In short, Dr. Carpenter argues that the cerebral substance manifests an elective affinity for alcohol in the blood: "the alcohol being thus specially drawn out of the circulating current by the nervous matter, and incorporated with its substance, in such a manner as even to change (when in sufficient amount) its physical as well as chemical properties." Its action is thus described by Dr. Carpenter:—

"The selective power of alcohol appears to lead it in the first instance to attack the cerebrum, the intellectual powers being affected before any disorder of sensation or motion manifests itself; and to this it seems to be limited in what has been here described as the first stage of intoxication. But with the more complete perversion of the intellectual powers which characterises the *second* stage, we have also a disturbed function of the *sensory ganglia*, upon which the cerebral hemispheres are superposed; this disturbance being indicated by the disorders of sensation, and also by the want of that control over the muscular movements which require sensation for their guidance. In the *third* stage, the functions of the cerebrum and sensory ganglia appear to be completely suspended, and those of the *medulla oblongata* and *spinal cord* now begin to be affected, as we see to be indicated by the difficulty of respiration, the strabismus, the dilated pupil, and the tetanic spasms."

There is considerable difference, however, in the temporary insanity and the other phenomena of morbid cerebral action induced by alcohol. It is certain that in some persons the influence on the motor system is much more manifest than on the sensorial, for in the class of cases to which we refer, while the individual sits still, he but slightly betrays his devotion to the glass, and it is only when he attempts locomotion that it is discovered he is too drunk to walk. This and other special states are referred to by Dr. Wilson.

"Sometimes a kind of reverie occupies the transition stage between that of excitement and complete intoxication, and the individual remains for a while in a state of simpering quiescence. With another, one solitary idea, generally some real or fancied subject of offence, seems to lay hold of all that is left of the intelligence, and he mutters his resentment with stolid perseverance. In some, the drunkenness sets in suddenly, after the drinking has been continued for a time previously without any marked indication of its effects; while in a few examples, the power of locomotion seems to be implicated to a greater extent than that of the intelligence, and the drunkard loses the faculty of rendering his movements co-ordinate, and reels and staggers in his gait, though he still retains an entire consciousness of his condition. Or there may be the contrary of this, which is not of unfrequent occurrence, where the staring, vacant eye, and the expressionless features, with the inarticulate speech, surprise one in an individual who can still walk with almost perfect steadiness, though with a peculiar air of indecision in his movements. In such instances, which, in common with most observers, I have repeatedly had occasion to remark, there are physiological grounds for believing that, in the first description, it is the cerebellum, or smaller division of the brain, which is chiefly affected; and in the latter, the cerebrum, or larger division."

Doubtless individuals differ widely as to the relative vigour of the various divisions of the nervous centres, and as to their susceptibility to assume a morbid condition on the application of morbific agents; a difference to which may be obviously attributed the variety in the phenomena of intoxication by alcohol. The congenital condition of the nervous system, the education and employments, and the addiction to other vices, as gluttony, debauchery, &c., are causes of fundamental differences. The length of period during which the brain has been subjected to the action of the poison, must exercise an important influence. It may be stated, in general terms, that the phenomena of intoxication are as varied as those of mental derangement.

We may with propriety here revert to another point in the natural history of drunkenness, namely, the dangerous adulteration of spirits and intoxicating liquors.* Poisonous ingredients may be added either wilfully or accidentally. In England, common malt liquors are rendered stronger, that is, more intoxicating, by the addition of *cocculus Indicus*. In countries where spirits are distilled from potatoes and the cereals indiscriminately, it is probable that they are adulterated with some of the nervine-irritants and acro-narcotic poisons common to a large number of *fungi*. Dr. Huss is of opinion that the brandy distilled from diseased potatoes contains some new principle, termed by the Swedish distillers "brännsnyta," which is not to be met with in spirit distilled from fresh potatoes or sound grain, and the operation of which is similar to that of alcohol. In Germany, a some-

* Vide Dr. Hassall's valuable work, recently published, "On Food, and its Adulterations." 1855.

what similar principle is obtained from distilled spirits, termed "fusel oil." The common *Lolium*, and the *Raphania raphanistrum*, (a weed growing in the corn-fields in Sweden and most parts of northern Europe,) are both poisonous. Linnæus, believing the latter to produce the kind of phenomena known as Ergotism, (that is, the results of poisoning by ergotted or spurred rye,) termed the disease *Raphania*. Amongst these phenomena are enumerated epilepsy, delirium, insanity, and idiotcy. Although the police in Germany interfere to prevent the sale of spurred rye for food, they do not prevent its use in distilling, nor the use of the poisonous cereals we have noticed. In fact, any vegetable matter capable of the saccharine fermentation, is used by distillers in Germany and the north of Europe—spurred rye, mildewed grain, bad potatoes, husks of grapes, &c. Now, all these have a very close connexion with microscopic *fungi;* and hence the probability, that the known poisonous principles of these minute mushroom growths are held in solution in these foreign kinds of spirits, and may be the true source of the acrid stupifying properties which they especially possess. Further, the fusel-oil itself is not pure, but contains metallic oxides of known virulent action on the nervous system. One specimen the concrete oil, when examined, was found to contain 32·3 per cent. of these oxides, namely:—22·5 oxide of copper, 6·3 oxide of tin, and 3·5 oxide of lead! How much of the poisonous principle derived from the lolium, or from the ergot, or from the poisonous fungi that constitute the deadly vegetation of the distillers' refuse, enters into the hideous compounds which the drunkard swallows hourly, it is not practicable to determine, nor is it of importance to our subject. Certain it is, that poisons of this kind are taken with the inferior spirits.

The action of opium, hasaich, and other drugs upon the nervous system, taken for the same purposes as alcohol, differs considerably from that of the latter agent. As regards the mental powers, opium seems to act almost exclusively upon those portions of the cerebral hemispheres which constitute the seat of the intellect and imagination. The "Confessions of an English Opium-Eater" contains an instructive comparison of the effects of opium and alcohol:—

"Crude opium, I affirm peremptorily, is incapable of producing any state of body at all resembling that which is produced by alcohol; and not in *degree* only incapable, but even in *kind;* it is not in the quantity of its effects merely, but in the quality, that it differs altogether. The pleasure given by wine is always mounting, and tending to a crisis, after which it declines; that from opium, when once generated, is stationary for eight or ten hours; the first, to borrow a technical distinction from medicine, is a case of acute—the second, of chronic pleasure; the one is a flame, the other a steady and equable glow. But the main distinction lies in this—that whereas wine disorders the mental faculties, opium, on the contrary, (if taken in a proper manner,) introduces among them the most exquisite order, legislation, and harmony. Wine robs a man of his self-possession; opium greatly invigorates it. Wine unsettles and clouds the judgment, and gives a preternatural brightness, and a vivid exaltation to the contempts and the admirations, the loves and the hatreds, of the drinker; opium, on the contrary, communicates serenity and equipoise to all the faculties, active or passive. . . . . . In short, to sum up all in one word, a man who is inebriated, or tending to inebriation, is, and feels that he is, in a condition which calls up into supremacy the merely human, too often the brutal, part of his nature; but the opium-eater (I speak of him who is not suffering from any disease, or other remote effects of opium,) feels that the diviner part of his nature is paramount; that is, the moral affections are in a state of cloudless serenity, and over all is the great light of the majestic intellect."

The influence of these nervine poisons is not limited, however, to the cerebral tissues. The entire nervous system participates in the morbid action, and consequently the spinal and sympathetic ganglia are also involved. As to these latter, opium and alcohol appear to have widely different relations, for the *immediate* influence of opium upon the viscera is almost exclusively sedative, of alcohol, stimulant. This difference of action shows itself also in a marked manner in the more *permanent* morbid changes induced by the two poisons; for opium finally exalts sensibility, alcohol abolishes it. These more permanent changes merit inquiry.

The constantly recurring action of a nervine stimulus, follows in its results on the appetite the law of habit; that is to say, it is at last a necessary stimulus, and is urgently desired, in the same way as food, drink, &c. But there is this difference between this morbid and a natural appetite for a stimulus—that when the latter is artificial and induces pathological changes, the need for it augments *pari passu* with the changes themselves. In habitual drunkenness, and in opium-eating, this is undoubtedly the case, although there are exceptional instances even as to them. The quantity of alcohol taken occasionally in these gradually increasing doses, is in some instances enormous. Dr. Farre mentioned to the Committee of Inquiry of the House of Commons, the case of a gin-drinker, "the largest man he ever saw," who had been known to drink seventy-two glasses of the usual drams at a sitting. Dr. Wilson mentions several similar instances. An inmate of the workhouse at Hanover had been in the habit of taking from half a gallon to a gallon of spirits almost every day. Chomel cites the instance of a patient aged thirty-four, who had been in the habit of drinking fifteen bottles of wine and four of brandy, daily. Esquirol knew another instance in which 171 *petits verres* of brandy was the daily consumption. The cause of this insatiable thirst for stimulants lies partly in the state of the blood and the nervous centres, partly in the morbid state of the stomach. In the absence of the alcohol from the former, there is in fact nothing to supply its place, as in ordinary health; while in the inflamed and irritated state of the gastric mucous surface, there is a direct excitant of the morbid sensations we have described as resulting from this cause. Hence that indescribable feeling of sinking and oppression, which renders life intolerable to the drunkard, until the nervous centres are again stimulated. As the action of the alcohol gradually abates, in consequence of its being used up or eliminated by the excreting surfaces, a fresh supply is continually taken to supply the waste, except during sleep. This cessation from the action of the stimulant during the night is the principal, though not perhaps the sole, reason why the nervous depression is the greatest, and the thirst for spirits so urgent, on awaking in the morning.

At this, the confirmed stage of alcoholic intoxication, the brain is diseased, and both the motor and intellectual powers are, for the most part, enfeebled. The sufferer (for such he emphatically is), is incapable of any prolonged bodily exertion or continuous thought, and the incapacity for fixing the attention may increase so as to amount to confusion of ideas. Spectral illusions are not unfrequent, even although there be no actual approach to delirium, and imaginary sounds and voices are heard. The moral and emotional feelings undergo a degradation progressing *pari passu* with the cerebral disease, so that the high-minded, honourable man has become a cunning, selfish liar or cheat, the religious man a sensualist, the faithful husband an adulterer, the indulgent father a ferocious tyrant and a constant terror to his family. German writers (as Clauss and Berndt) take special note of this change in the moral character of the drunkard, designating it *Inhumanitas ebriosa.* They distinguish two principal forms, namely, *Ferocitas ebriosa* and *Morositas ebriosa;* the former is seen in men of powerful frame, is characterised by brutal violence, and often ends in furious mania; the latter is seen in individuals of a more delicate organization, following sedentary employments, and is apt to end in melancholia or suicide. Nothing is more certain in the progress of intoxication than this moral degradation. The history of drunkenness abounds with illustrations of the general principle so striking that they would be incredible if not confirmed by daily observation. We find the following in the Parliamentary Report of 1834. A widow, the aunt of a most celebrated and distinguished vocalist, fell into habits of gin-drinking and wasted her fortune. One of her sons was in the employ of Mr. Samuel Herapath, (who relates the history,) and lodged with a poor woman. He happened to go home to his wretched mother one Saturday night, and the consequence was, that while he was asleep she robbed him of his earnings, and pawned his shirt and coat to spend all in drink. The boy being ashamed to go back to his employer, she persuaded him to turn pickpocket, and he was ultimately transported. The same woman had actually taken every tooth out of her head except two, and sold them, so as to be able to purchase gin; and she would have sold these also, but she could only get fourpence for the last one she had sold. Mr. Poynder mentioned to the Parliamentary Committee the instance of a man of the name of Smith, a drunkard, who was tried for setting fire to his house, in Newgate-street, and whose wife died almost immediately after he was suspected of doing it. The jury acquitted him on the ground that it was possible his wife (also a drunkard) had done it. On his death-bed he confessed that he actually had induced her to set fire to the house, and had poisoned her as soon as suspicion fell upon him, least she should betray his secret. Ferocious crimes of every kind, prostitution, and the lowest licentious indulgence are also amongst the moral degradations of the drunkard. Domestic virtue and happiness are utterly annihilated. Mr. Broughton mentioned to the Parliamentary Committee an instance of a family, the father and mother of which were both habitual drunkards. The father was a respectable mechanic, and, in addition to earnings of two guineas a-week, might have had an income from property that came to him by will, of 200*l.* a-year. Yet his home was worse than a dog-kennel: it was one room; there was no bed, only a few old rags in a corner, into which his four children huddled; all occasions of nature in both ways were done in the room; and it was quite clear, from inspection, that for the common purposes of nature they never went anywhere else. As to the development of the ferocious characteristics of man by drunkenness, the police reports in the newspapers are full of the most painful examples. Brutal violence to the nearest and dearest relatives is a frequent result of alcoholic poisoning.

The transition from this degradation of the moral and intellectual faculties, as the result of morbid cerebral action, to actual and acknowledged *insanity*, is but a step. All persons experienced in the treatment of this disease are well conversant with the general fact, that drunkenness is amongst the more common causes of mental derangement, not only, indeed, by the direct morbific action of alcohol on the encephalon of the individual, but also by the transmission of a special constitution of the nervous system (thus acquired) to his offspring, which renders them peculiarly liable to nervous affections of every kind, but more particularly to various forms of insanity, amongst which may be specially mentioned that uncontrollable desire for stimulants, termed *oinomania*. *Delirium tremens*, or the drunkard's delirium, is, in fact, an acute paroxysm of mania running its course quickly, and it is only in the acuteness of its progress, and the intensity of its symptoms, that it differs from *mania ebriosa*, or *à potu*—a real insanity.

This latter affection appears under various forms, and is relatively of frequent occurrence. In the statistical tables contained in the Report of the Commissioners of Lunacy for 1844, illustrating the etiology of insanity, 15 per cent. of the cases then under treatment were attributed to drunkenness. Dr. Carpenter justly observes, that of 4·6 per cent., in which it is attributed to vice and sensuality, an excessive use of alcoholic liquors must have shared. Moreover, in every case in which hereditary predisposition was traceable, that alone was mentioned, although it is certain that such predisposition may remain dormant altogether, if not excited into action by habitual drunkenness. It is probable that, at the lowest, the proportion of one-fourth, or 25 per cent., of all cases of insanity may be attributed to habitual intoxication, considered both as an exciting and predisposing cause. This ratio will necessarily vary, however, according as the general population is more or less given to drunkenness. In the Report of the Commissioners, the proportion assigned to intemperance of the patients in nine provincial private asylums, is 32½ per cent.; while, according to Macnish, of 286 lunatics in the Richmond Hospital, Dublin, one-half were drunkards. Parchappe states that 28 per cent. of the cases at Rouen were due to drunkenness. At Turin, Bonacossa found the proportion of drunkards to be 22 per cent. males, and 2 per cent. females; in Holland, 11 per cent. males, 1 per cent. females. In Berlin, every third case of lunacy among the lower classes is the result of intemperance. Habitual dram-drinking is more prevalent in northern than in southern Europe, and so is insanity. In Italy the lunatics are in the proportion of 1 in 3785 of the population; in England, Sweden, Scotland, Denmark, Norway, the proportion is 1 in 783, 770, 575, 532, and 309, respectively. In our first volume we gave a table (p. 314) of the relative proportions of insane persons in Norway in 1825 and 1835, after the spirit-duty had been abolished for ten years. The increase, allowing for the increase of population during the *decennium*, was, in the towns, 32·9 per cent., in the rural districts, 69 per cent. In the various forms of insanity, the increase was, as to mania, 41 per cent.; melancholia, 69 per cent.; dementia, 52 per cent.; but most striking of all, as showing the influence of drunken parents in the cerebral development of their offspring, *congenital* idiotcy had increased 150 per cent.! In 1825, before an impulse had been given to the use of spirits by an abolition of the duty, the congenital idiots were only in a proportion a little more than one-third of the whole lunatic population; in 1835 they were nearly one-half. Dr. Howe alleges as a fact, having a similar explanation, that of 300 idiots in the State of Massachusets, whose history he investigated, 145 were the children of intemperate parents. That dram-drinking is the probable cause of this large increase (as Professor Holst, to whom we owe these statistics, affirms) is also shown by another consideration. Drunkenness is a more frequent vice amongst men than women. There entered, during one week of 1834, into fourteen gin-shops in London, 142,453 men, and 108,593 women—a great disparity, but greater if it be remembered that probably a large number of the women went to bring spirits home for their husbands. Now woman, in virtue of her special constitution, is really more predisposed to cerebral disorder than man, yet in Norway we find the proportion of male lunatics greater than of females, in all forms of derangement except melancholia. The proportion of males was 1 in 1449; of females, 1 in 1763. The preponderance of melancholic cases in the female population is the reverse of these proportions, and may perhaps be fairly attributed, in some degree at least, to the domestic misery which habitual drunkenness of the father induces in a family. Other northern countries exhibit the same coincidence between the prevalence of insanity and drunkenness. In Sweden, where the lunatics are in the proportion of 1 to 770 of the population, Professor Huss states that about half the number of insane males have been intemperate. Of from sixty to seventy men received into the asylum at Stockholm (we quote from Dr. Wilson), only ten were insane from other causes than drunkenness. In the great asylum at St. Petersburg (the Russians are notoriously a drunken people), out of 997 admitted during ten years, 837 were rendered insane, directly or collaterally, by intoxication. The specific forms of insanity which alcoholic poisoning develops may be classified under two or three principal heads. Firstly, there is the temporary or acute mania, known as *delirium tremens;* secondly, that general loss of mental power known as dementia or imbecility; thirdly, the destructive maniacs and monomaniacs, more especially the homicidal; and fourthly, the perversions of the instinct for life and physical well-being, melancholia, and suicidal monomania. The homicidal fury of drunkenness, and the homicidal impulse which the vice excites, are too well known to need special notice; nor need we dwell upon the cases of the demented and imbecile; the suicidal form is the most instructive.

Dr. Wilson distinguishes two forms of *suicidal mania* in the drunkard. In the one there is an exercise of the reasoning powers; in the other, the development of a blind impulse. The reasoning drunkard who commits suicide, stung by remorse and shame, premeditates the deed. Dr. Wilson remarks:—

"Everything reproaches him. His bodily pains, his waning vigour, his mental chagrin, his feelings of shame and repentance, yet his inaptitude for reform; his failure, not only in his duties towards society, but his habitual outrage of its purest principles, perhaps his loss of fortune and the ruin of his family, are all sources of perpetual agony; and he has besides systematically deprived himself of the best sources of consolation. It is in this condition that the drunkard, sinking deeper and deeper into despondency, begins to contemplate the possibility of terminating his evils, in at least as far as this world is concerned, at a single stroke; and brooding incessantly over his purpose, and carefully maturing its design, at last, in some moment of more than ordinary desperation, or during the shame and depression consequent on some more than ordinary excess, the fatal blow is struck."

The unpremeditated form of suicide is usually observed in the very paroxysm of intoxication, and seems to be a blind impulsive act, analogous to the blind ferocity of the drunkard, the result of that morbid action which alcohol excites within the encephalon.

These statements will suffice to illustrate the cerebral pathology of drunkenness; we will now turn to that of *opium-eating*. The public attention has not been drawn so strongly to this destructive habit as to that of intoxication, partly because the baneful effects are less public, partly because they are less injurious to the individual and to society—we say *less*, only because the evil effects of alcoholic intoxication are literally incalculable. It is well known, however, that the practice of opium-eating is much on the increase.

The pleasure induced by opium is dependent, almost exclusively, as we have already observed, upon its action upon the cerebral hemispheres. Its first influence is to refine and exalt the imagination and the intellect. The "English Opium-Eater" denies that it produces, of necessity, inactivity or torpor.

"Yet, in candour, I will admit that markets and theatres are not the appropriate haunts of the opium-eater when in the divinest state incident to his enjoyment. In that state crowds become an oppression to him; music, even, too sensual and gross. He naturally seeks solitude and silence, as indispensable conditions of those trances, or profoundest reveries, which are the crown and consummation of what opium can do for human nature. * * * Oh! just, subtle, and mighty opium! that to the hearts of poor and rich alike, for the wounds that will never heal, and for 'the pangs that tempt the spirit to rebel,' bringest an assuaging balm; eloquent opium! that with thy potent rhetoric stealest away the purposes of wrath; and to the guilty man, for one night, givest back the hopes of his youth, and hands washed from blood; and to the proud man, a brief oblivion, for

'Wrongs unredressed, and insults unrevenged;'

that summonest to the chancery of dreams, for the triumphs of suffering innocence, false witnesses; and confoundest perjury; and dost reverse the sentence of unrighteous judges;—thou buildest upon the bosom of darkness, out of the fantastic imagery of the brain, cities and temples, beyond the art of Phidias or Praxiteles—beyond the splendour of Babylon and Hekatómpylos; and, 'from the anarchy of dreaming sleep,' callest into sunny light the faces of long-buried beauties, and the blessed household countenances, cleansed from the 'dishonours of the grave.' Thou only givest these gifts to man; and thou hast the keys of Paradise, oh, just, subtle, and mighty opium!"

Such is the Anacreontic prose of the gifted author of "The Confessions," in describing the primary psychological effects of opium. Its action on the hemispherical ganglia is to excite the phenomena of dreaming, both in sleep and in waking, and to virtually suspend the influence of the will on the organ of mind. This result is attained partly by its direct action on the latter; partly, probably, by its action on the sensorial ganglia, and on the sensational periphery, in virtue of which it arrests or obtunds those multitudinous impressions which flow upon the sensorial centres from the organs of special sense, all parts of the skin, and mucous surfaces of the viscera, and which, by their continuous but varying operation, modify the states of consciousness at every moment, through the varied changes they induce on the ultimate structure of the vesicular neurine of the brain. The external world is, in fact, in so far shut out that it cannot reach the will, and operates no further than the cerebrum. It is from this continued morbific operation of opium upon the sensorial system that the *sufferings* of the confirmed opium-eater originate.

Opium, like alcohol, must be taken in continually increasing doses to produce the desired effects, and, when taken to a certain point, it also, like alcohol, becomes an imperious necessity, to which everything in life must bend. The quantity taken in a day by confirmed opium-eaters seems incredible. The "English Opium-Eater" took 320 grains per day, *i.e.*, 8000 drops of laudanum—according to his own estimate, 80 teaspoonsful—or what would amount to about *ten ounces* of laudanum; but this is little more than one-fourth of what Coleridge took on one occasion in the twenty-four hours—namely, a whole quart! Indeed, he had been long in the habit of taking from two quarts of laudanum in a week to *a pint* a-day.

The operation of continuous opium-eating is, like that of alcohol, to degrade and enfeeble the moral and intellectual faculties, as well as the bodily powers. Dr. Oppenheim thus describes the Turkish victim of the drug:

"The habitual opium-eater is instantly recognised by his appearance. A total attenuation of body, a withered, yellow countenance, a lame gait, a bending of the spine, frequently to such a degree as to assume a circular form, and glassy cheeks, sunken eyes, betray him at the first glance. The digestive powers are in the highest degree disturbed; the sufferer eats scarcely anything, and has hardly one evacuation in a week; his mental and bodily powers are destroyed; he is impotent. * * * After long indulgence the opium-eater becomes subject to nervous or neuralgic pains, to which opium itself brings no relief. These people seldom attain the age of forty, if they have begun to use opium at an early age. * * * When this baneful habit has become confirmed, it is almost impossible to break it off; the torments of the opium-eater, when deprived of this stimulant, are as dreadful as his bliss is complete when he has taken it; to him night brings the torments of hell, day the bliss of Paradise."*

The "English Opium-Eater" vividly describes the loss of all power of the will and of intellectual effort, which are the morbid results of the drug.

"But for misery and suffering, I might, indeed, be said to have existed in a dormant state. I seldom could prevail on myself to write a letter; an answer of a few words to any that I received was the utmost that I could accomplish; and often not *that*, until the letter had lain weeks or even months on my writing-table. * * * The opium-eater loses none of his moral sensibilities, or aspirations; he wishes and longs, as earnestly as ever, to realize what he believes possible, and feels to be exacted by duty; but his intellectual apprehension of what is possible infinitely outruns his power, not of execution only, but even of power to attempt. He lies under the weight of incubus and nightmare; he lies in sight of all that he would fain perform, just as a man, forcibly confined to his bed by the mortal languor of disease, who is compelled to witness injury or outrage offered to some object of his tenderest love:—he curses the spells which chain him down from motion; he would lay down his life if he might but get up and walk; but he is powerless as an infant, and cannot even attempt to rise."

Alcohol acts upon that portion of the hemispherical ganglia which is the organ of the representative faculty, and in *delirium tremens* excites the wildest phantasmagoria. So also opium, but perhaps less coarsely, or with grander imagery. As the English opium-eater lay awake in bed, vast processions passed along in mournful pomp, friezes of never-ending stories, that to his feelings were as sad and solemn as if they were stories drawn from times before Œdipus or Priam, before Tyre, before Memphis. His dream partook doubtless of the character of his imagination, which was filled, amongst other things, by oriental imagery, and impressed "unimaginable horrors" upon him. "I seemed every night to descend, not metaphorically, but literally to descend, into chasms and sunless abysses, depths below depths, from which it seemed hopeless that I could ever re-ascend." The states of gloom which attended the gorgeous spectral phenomena of his

* "Ueber den Zustand der Heilkunde und über die Volkskrankheiten in der Europäischen und Asiatischen Turkie. Ein Beitrag, &c. Von Fried: W. Oppenheim (1853), p. 93.

sleeping state amounted "at least to utter darkness, as of some suicidal despondency, not to be approached by words." De Quincey's descriptions of his dreaming phenomena is "a very interesting addition to mental pathology." We subjoin one of many illustrations:—

"I was stared at, hooted at, grinned at, chattered at, by monkeys, by paroquets, by cockatoos. I ran into pagodas, and was fixed for centuries at the summit, or in secret rooms; I was the idol, I was the priest, I was worshipped, I was sacrificed; I fled from the wrath of Brama through all the forests of Asia; Vishnu hated me; Seeva laid wait for me. I came suddenly upon Isis and Osiris; I had done a deed, they said, which the ibis and the crocodile trembled at. I was buried for a thousand years in stone coffins, with mummies and sphinxes, in narrow chambers at the heart of eternal pyramids. I was kissed with cancerous kisses, by crocodiles, and laid confounded with all unutterable slimy things, amongst reeds and Nilotic mud."

This "cursed crocodile" became at last an object of more horror than all the rest. The abominable head of the beast and his leering eyes looked at him, multiplied a thousand times, and he stood loathing and fascinated.

The corresponding action of alcohol has been vividly described by one who has experienced its terrors, and the phenomena are interesting in comparison with the preceding.

"Hideous faces (Mr. J. B. Gough remarks in his 'Autobiography') appeared on the walls, and on the ceiling, and on the floor; foul things crept along the bed-clothes, and glaring eyes peered into mine. I was at one time surrounded by millions of monstrous spiders, who [which] crawled slowly, slowly, over every limb, whilst beaded drops of perspiration would start to my brow, and my limbs would shiver until the bed rattled again. * * * * And then the scene would change. I was falling—falling—swiftly as an arrow, far down into some terrible abyss; and so like reality was it, that as I fell I could see the rocky sides of the horrible shaft, where mocking, gibing, mowing, fiend-like forms were perched; and I could feel the air rushing past me, making my hair stream out by the force of the unwholesome blast."

The operation of continued opium-eating on the sensorial system is to develop its susceptibilities so that all ordinary impressions are painful and irritating so soon as the drug ceases to be taken. The "English Opium-Eater" thus describes the sufferings he experienced when he resolutely emancipated himself from the tyranny of the drug:—

"Meantime, the symptoms which attended my case for the first six weeks of the experiment were these:—Enormous irritability and excitement of the whole system; the stomach in particular restored to a full feeling of vitality and sensibility; but often in great pain; unceasing restlessness night and day; sleep—I scarcely knew what it was; three hours out of the twenty-four was the utmost I had, and that so agitated and shallow, that I heard every sound that was near me; lower jaw constantly swelling; mouth ulcerated; and many other distressing symptoms that would be tedious to repeat, amongst which, however, I must mention one, because it had never failed to accompany any attempt to renounce opium,—viz., violent sternutation; this now became exceedingly troublesome, sometimes lasting for two hours at once, and recurring at least twice or three times a day. * * * * I protest to you that I have a greater influx of thoughts in one hour at present, than in a whole year under the reign of opium. It seems as though all the thoughts which had been frozen up for a decade of years by opium, had now, according to the old fable, been thawed at once—such a multitude stream in upon me from all quarters. Yet such is my impatience and hideous irritability, that, for one which I detain and write down, fifty escape me; in spite of my weariness from suffering, and want of sleep, I cannot stand still or sit for two minutes together."

Another form of drunkenness remains to be described,—namely, the *paroxysmal.* This is the form which has been mentioned by writers (first by Hufeland, who termed it *Dipsomania*) as a true mania, and which is recognised to be such by all practically acquainted with insanity. Erdmann first observed this affection in Russia, where it is termed *sapoi* (sauf-sucht, drinking disease, or mania). Brühl-Kramer, Erdmann, Friedreich, Henke, Guislain, and others, have also treated of it. Broussais and Rayer adopted the term *Oinomania.* Many writers have, however, treated of the affection as if it were a form of delirium tremens, to which it is undoubtedly generically allied, but from which, nevertheless, it is specifically distinct. Persons affected with the *paroxysmal* form are for the most part of temperate or even abstinent habits, and are only attacked at intervals with the disorder, which consists in the gratification of an impulse to swallow stimulants in enormous doses for a period of definite duration, when the paroxysm ceases and the individual resumes his temperate or abstinent mode of life. Dr. Hutcheson, of the Glasgow Lunatic Asylum (Report for 1842), has given the best detailed account of the disease in the English language. He notes three forms,—the acute, the periodic, and the chronic. The acute is the rarest of the three, and occurs as a sequel of exhausting causes, as fevers, puerperal or uterine hæmorrhage, excessive venereal indulgence, &c., or in certain forms of dyspepsia; in the latter case it is very apt to become chronic. The periodic form is met with in persons who have experienced injury of the head, or who have overworked the brain, or who are the offspring, directly or collaterally, of drunkards or lunatics. Women are apt to become the subjects of it during pregnancy. The *chronic* is simply the paroxysmal form changed into *continuous* drunkenness.

When a person is about to have a paroxysm of *oinomania*, and it is not induced by any manifest excitant, as alcohol, fatigue, &c., he feels listless, uneasy, restless, and depressed, and is incapable of steady application. These feelings are accompanied by a gradually increasing craving for stimulants, which at last is yielded to. The individual, perhaps, then disappears from his home or usual place of business, and spends his days and nights in alternate sleep and intoxication, haunting the lowest dram-shops, and associating with depraved persons. Or perhaps he shuts himself up in his room, never leaving it for any purpose, and rapidly gulps down glass after glass of liquor he has procured, reckless of all consequences to himself, his family, or his affairs. The paroxysm being exhausted, a stage of apathy and depression succeeds, in which bitter regrets for his folly, and resolutions never again to yield to temptation, are prominent. This period of temperance may continue for some months, when, after an apparently trivial circumstance, the morbid cerebral condition which constitutes the paroxysm is again developed.

Friedreich notes five stages of the affection, as follows:—1. The *premonitory* stage. After a period of apparent health, and moderate use of stimulants, the eyes present a wild expression, there is spasmodic action of the muscles of the orbit, a winking of the eyelids, photophobia, flushing of the face, headache, disturbed sleep, loss of appetite, indigestion, flatulence, anxiety, and dread. This stage continues for from a few hours to a few days. 2. The *commencement* of the attack. Increased desire for spirituous drinks, which relieve the restlessness for a short time, and to this end the patient takes them, but always more and more rapidly. 3. Stage of *development*. The desire for spirits is now more than ever urgent, and the relief given by them less in time and extent; if the attempts to take them be forcibly resisted, so that the supply is cut off, the want is immediately followed by great distress, and feelings of anguish, fainting, and suffocation; indeed, not unfrequently persons thus deprived of the desired stimulants became actually insane or maniacal. 4. *The crisis* occurs in 3, 5, 7, 9, 11, 13, or 21 days. It is characterized by feelings of intense distress, so that the patient loudly bewails his state, or groans deeply, until at last urgent vomiting supervenes, when either "corrupted" bile, or in many cases a watery fluid, is thrown up. To this succeeds the greatest disgust for spirituous drinks, so that the person who but a short time before urgently demanded brandy, now shudders at the bare idea of it. 5. The stage of *convalescence* is marked by the *sequelæ* of the affection, amongst which an excited condition of the entire system is the principal. There are also sleeplessness, frightful or disagreeable spectral illusions, and depressing and distressing sensations,—the phenomena more or less, in short, of *delirium tremens.*

The leading symptoms in the typical form of the disease are those which show themselves in the thoracic viscera in connexion with the appetite for stimulants,—namely, the feelings of anguish, restlessness, and impending death by suffocation, and those which are more purely mental, and in which the insatiable appetite is the most prominent. To these may be added the direct results of the alcoholic poisoning. In discussing the pathology of paroxysmal drunkenness, it is necessary to determine carefully the order of causation. Now, it is undeniably certain that in every case, whether it be acute or periodic, there is a special condition of the cerebrum which predisposes the individual to the paroxysm. This may be termed the *predisposing* cause. Without this, those circumstances upon which the outbreak immediately supervenes, or in other words, the *exciting* causes, could never take effect. The *proximate* cause is that condition of the cerebrum which is developed by the exciting causes in a person duly predisposed, which condition is necessary to the manifestation of the paroxysm. The operation of these causes is best illustrated by cases. A member of a liberal profession is subject to paroxysms of oinomania. He is fully aware of his infirmity, and is a water-drinker on principle; for, so long as he abstains from alcoholic stimuli, he is safe. If, however, he yields to temptation ever so little,—if he takes but a single glass of wine,—he is lost. The irresistible appetite is excited, and all the misery and disgrace of a paroxysm of drunken madness follows. This individual has a near blood-relative, a man of superior talents, who is equally predisposed to oinomania, and who, when attacked by a paroxysm, disappears from his family and home, and is found in the lowest haunts of vice and depravity, drinking with the most depraved. Both these examples are members of a family in which insanity is hereditary. In another similar case of an individual—a member of an artistic profession—there is great natural talent and aptitude for business, so that he gives the highest satisfaction to his employers; but at varying intervals of time—from a few weeks to several months—the oinomaniac is absent from his office for several days on a drunken "spree." When he returns, great is his remorse, bitter his self-condemnation, loud and resolutely expressed his promises to resist temptation. For a while all goes on well; but, sooner or later, the temptation comes, the alcoholic stimulant is presented, is irresistible, and a paroxysm is the result, to end as before. Now the *brother* of this impulsive oinomaniac is the victim of continuous drunkenness; the *father* of both was a continuous drunkard, who believed himself to be a tea-pot, to be made of glass, &c., and who, in a paroxysm of inebriate fury, burnt a cat alive; and the *grandmother's* brother was also an impulsive and finally a continuous oinomaniac. It is related of this grand-uncle, that his friends having taken away his clothes on a Sunday morning, hoping to confine him to the house by the want of clothing, he went into his warehouse, and donning a funeral-cloak, made his way to the dram-shop! These cases illustrate the *hereditary* transmission of the predisposition from generation to generation.

Like insanity, epilepsy, and other analogous affections of the cerebrum, oinomania may be *periodic*. Brühl-Cramer mentions a case in which the paroxysm occurred regularly every four weeks, at the new moon, and Most remarks that he thinks he has observed in several instances that the impulse to drink was the most urgent about the same time. In Henke's "Zeitschrift für Staatsarzneikunde (vol. 34), a case is related of monthly periodic drunkenness prolonged for seven years; each attack occupied eight days. The patient was a mechanic; orderly, industrious, and moral, until he was thirty-four, when he became subject to paroxysms of oinomania, during which his whole character underwent a change. After being for three weeks most industrious and steady, he would return home of an evening in apparently his usual health, but on going to bed he could not sleep on account of great depression and a peculiar sensation in the head. About one o'clock he would leap out of bed, run about the house, rush into the street, in nothing more than his shirt, and shout and rave so violently for spirit at the dram-shops, that the people were compelled to supply him; this he would drink greedily and in large quantities, until he lost the use of his limbs. Towards morning he would be taken home unconscious, where he would be confined and bound. After lying in that state, with half-closed eyes, for a length of time, he would raise himself up, look round with a wild, melancholy look, the veins of the forehead starting, his face bathed in perspiration, his pulse quick and full, his hair dishevelled, his body almost naked: he would first be abusive, twist about, and make violent efforts to free himself from restraint, and then would piteously beg and implore for spirits, his voice gradually becoming weaker. He rejected all food and drink except coffee, demanding brandy only, for without it he felt he must perish. He was usually given to drink, for the purpose of quieting him, brandy-and-water, in the proportion of one of brandy to three of water, which he would drink off with the utmost eagerness, and immediately ask for more. In this way he would go on without resting or sleeping for one moment for eight days, having brandy-and-water given to him two or three times a day, and taking hardly anything else. During this time he became gradually weaker, and his voice more and more feeble, and at last he would fall asleep, exhausted. On awaking, he had no recollection of what had happened, felt weak, and trembled a good deal. The appetite for food then returned; he would drink water only, abhorred brandy, went back to his employment, and was an industrious, steady, temperate man until the next paroxysm. This would return at the regular period, whether he took brandy or not, and continued whether his desire for brandy was gratified or not. As years went on, the duration of the paroxysms became gradually shortened to six, five, and four days. There was no very striking decay of the intellect, although at last the termination of the case in imbecility began to threaten. He died unexpectedly during a paroxysm on the third day, appearing as if he had fallen asleep. During the paroxysms, his room was more like that of an insane person than of a rational being, had a very offensive smell, and was very filthy. The patient himself, also, looked like a maniac. The father of this man was a confirmed drunkard, and committed suicide by hanging; two of his brothers were drunkards,—only a sister and himself of the family remained free from the vice; and he showed no symptoms of oinomania until he was thirty-four.

This case illustrates the disease in the acute form described by Friedreich, and is specially interesting, inasmuch as by the character of regular periodicity which it presented, it brings oinomania into the general category of cerebral and cerebro-spinal affections, the majority of which are thus periodic. It will occur at longer intervals, however, than the month, just as mania, epilepsy, somnambulism, &c., will. Cases continuing for one week, and recurring at intervals of twelve weeks, have been observed.* In the first case which Guislain saw, the paroxysm occurred at still longer intervals; it was that of a music-master, who every year, or every two years, suddenly ceased to practise his profession, and for about three months would be continually intoxicated. The paroxysm would then suddenly cease, and the patient become scrupulously temperate, drinking nothing but water, and avoiding all chances of temptation. Feeling during one of these lucid intervals the premonitory symptoms of a paroxysm, he committed suicide. In another case (a woman) mentioned by Guislain, the paroxysms came on after lucid intervals of from three to four years.

There are instances in which the affection seems to be analogous to that strange perversion of the appetite termed *pica*, which is seen in pregnant or hysterical women, or in persons affected with chronic malarious disease, as the dirt-eating negroes. In these cases there is the same irresistible appetite for some extraordinary article of diet, as in

* Most's "Ausführliche Encyclopädie der gesammten Staatsarzneikunde," vol ii. p. 995.

the oinomaniac for stimulating drinks, constituting in some a true monomania. Dr. Elliotson used to mention in his lectures as "an absolute fact," that a patient of this kind "has longed for raw flesh, and even for live flesh." The Messrs. Griffin had a young lady under their care of very delicate habit, who had been for a length of time suffering from oppression and constriction of the chest, hysterical fits, troublesome palpitations, and spinal tenderness, all which symptoms were aggravated once on a time, when she was at the sea-side for change of air. A blister was applied over the upper dorsal vertebræ, as far down as the eighth or ninth, with the object of relieving these symptoms, the operation of which was followed by an insatiable thirst, so that she drank a whole bottle of ale in a few minutes, besides wine, which she asked for repeatedly. She rested that night. The sequel we subjoin in the words of the mother of the patient.

"The next day at dinner she ate boiled mutton, drank a bottle of ale, and said that nothing but wine and ale would satisfy her. She had an hysterical fit of crying, but soon became calm; and seemed fairly that evening, except for the pain in her side, which, she said, nothing but eating relieved. After tea she went to bed, and asked for an egg and ale for supper; this she got, and asked for another. * * * During that night she got seven glasses of wine and camphor julep. At length I positively refused her any more, and entreated her to be still and calm; for she was frightfully impatient, talking incessantly, and begging for wine and ether. She had no oppression, but had the palpitation that night, and very much the following day. Her stomach at last grew sick, and she discharged it, throwing off much bile: she seemed better afterwards, and grew a little composed; next day I fed her thirst with slops and broth; she was exceedingly ravenous. She is now much better."*

The Messrs. Griffin, in commenting on this case, remark that the patient, in her general state of health, had a very slight appetite, and was never accustomed to more than the smallest quantity of wine or ale at any time. They think the state described to be connected with a feeling of nervous sinking, which is relieved by anything taken into the stomach. It is an interesting example of the *acute* form of oinomania. Nothing is said of the hereditary predisposition in this case, but, from the hysterical diathesis, and the peculiarity of the symptoms, one might infer *à priori* descent from a line of ancestors who had taken alcoholic stimulants unduly.

Women are not unfrequent subjects of the disorder; in two examples that have come under our notice of the recurrent or paroxysmal form in women, there were the usual symptoms of *gastritis*. The

* "Observations on Functional Affections of the Spinal Cord," &c. &c. By W. and D. Griffin, p. 52.

attacks were always ushered in by an intolerable feeling of distress about the epigastrium, amounting, sometimes, almost to a sensation of impending death. It was not easy to determine whether, in these particular cases, the gastric affection stood to the oinomania in the relation of cause or of effect; but we have seen one or two examples of incipient continuous drunkenness in which the former undoubtedly preceded the latter, and we are inclined to think that a chronic condition of the digestive organs in which this sinking sensation is a prominent symptom, and which is speedily relieved by a little hot brandy and water, or negus, is a not unfrequent cause of habitual intoxication in the sex. German writers designate it a *gastromalacia*, and have advocated the view that the spleen is deeply involved in the disorder. This is a feasible theory, for it is very certain that changes in the nutrient materials or composition of the blood in connexion with the supply of food and liquids, are amongst the most common antecedents to the outbreak of uncontrollable appetites.

The preceding cases and comments will amply suffice to illustrate the general pathology of this remarkable form of insanity, and it only remains for us to determine its psychological relations with a view to treatment. In the first place we may remark, that the mental condition of the oinomaniac is analogous to that in man and lower animals, in which there is an uncontrollable instinctive appetite developed, and the intellectual and moral faculties cease to act. The states of *extreme* hunger and thirst, either conjoined or occurring separately, are characterized by this uncontrollable impulse in irrational animals, and in men whose power of self-control is feeble. So also often the appetite for the natural food is impulsive, as when a carnivorous animal sees or smells his prey, or, even, only smells or tastes blood; or when herbivorous animals perceive that on which they thrive best, after having been long deprived of it. The instincts in relation with the reproduction of the species are equally impetuous, equally uncontrollable in lower animals as the appetite of the oinomaniac for stimulants.

> "Nonne vides ut tota tremor pertentet equorum

> Corpora, si tantum notas odor attulit auras?

> At neque eos jam fræna virum, neque verbera sæva,

> Non scopuli rupesque cavæ, atque objecta retardant

> Flumina, correptos nudâ torquentia montes."

Concurrently with this morbid development of an appetite there is a cessation or *diminution of the action of the will.* This is a very important point in the history of oinomania, especially in relation to those forms which are clearly to be traced to hereditary transmission, either from insane parents or from those who have enfeebled their cerebrum by nervine stimulants. Indeed, this infirmity of the will is itself virtually a species of imbecility, not always, doubtless, accompanied by imbecility of intellect, but, on the contrary, occasionally associated with the highest powers of thought and imagination. We know of no more interesting illustration of this general fact than the history of the two Coleridges, father and son. David Hartley Coleridge was born on 19th September, 1796, a date probably antecedent to that at which his father began to take laudanum, but we have ample evidence that about this time his father's temperament and mental state were very similar to his own. Thus, at the end of 1795 or beginning of 1796, Samuel Taylor Coleridge writes, "I am almost heartless! My past life seems to me like a dream, a feverish dream! all one gloomy huddle of strange actions, and dim-discovered motives! Friendships lost by indolence, and happiness murdered by mismanaged sensibility!"* There are also abundant illustrations of his irresolute will about this date. We have seen how completely the father surrendered himself to the practice of opium-eating; great was that father's distress, nevertheless, when his son lost his fellowship at Oriel College, Oxford, by intemperance, an infirmity which beset him through life. The habitual procrastination and irresolution of Samuel Taylor Coleridge re-appeared in his son Hartley with a difference, but in common with other leading mental characteristics of the father. His brother describes, in vigorous outline, the character of a man who abhors pain as he would death, and loves pleasure as he would life, when he depicts Hartley Coleridge as he was in childhood, and foreshadowed the "coming cloud."

"A certain infirmity of will had already shown itself. His sensibility was intense, and he had not wherewithal to control it. He could not open a letter without trembling. He shrank from mental pain; he was, beyond measure, impatient of constraint. * * * He yielded, as it were, unconsciously, to slight temptations, slight in themselves, and slight to him, as if swayed by a mechanical impulse apart from his own volition. It looked like an organic defect—a congenital imperfection."*

In short, Hartley Coleridge was unsuccessful in life, because, to use his brother's words, "he had lost the power of will." Of this he was himself aware, as is proved by some lines he wrote in a copy of his poems, in allusion to his *intention* of publishing another volume.

"Oh! woeful impotence of weak resolve
Recorded rashly to the writer's shame,
Days pass away, and Time's large orbs revolve,
And every day beholds me still the same,

* Cottle's "Early Recollections," vol. i. p. 170.

† "Poems by Hartley Coleridge. With a Memoir of his Life." By his Brother, vol. i. p. lix. (preface).

Till oft neglected purpose loses aim,
And hope becomes a flat unheeded lie."

Individuals with this peculiar infirmity of will, and this engrossing appetite for pleasure, manifest, occasionally, when in connexion with the predisposition to oinomania, a tendency to pursue, at intervals, a vagabond life. So we find it was with Hartley Coleridge, who (we are informed) had "a habit of wandering and concealment, which returned upon him at uncertain intervals during the middle portion of his life, exposing himself to many hardships, if not dangers, and his friends to sore anxiety." This state of mind is, by no means (as we have seen), an unusual symptom or phenomenon of oinomania itself.

Hartley Coleridge's character illustrated another peculiarity of the class of men we are considering, namely, their tendency to painful and distressing feelings, in alternation with an opposite state; and here, again, he shall describe his own mental condition in this respect.

"Sometimes, as if with mocking guile,
The pain departs a little while;
Then I can dance, and sing, and smile
With merry glee.

But soon, too soon, it comes again,
The sulky, stifling, leaden pain,
As a black cloud is big with rain,
Is big with woe.

All I ask is but to know
The depth and nature of the woe;
I hope not for a wind to blow
The cloud away.

I hear an inarticulate sound,
Wherein no fixed sense is found,
But sorrow, sorrow without bound
Of when or where."

Hartley Coleridge's brother remarks that this kind of temperament constitutes the "humourist," and "is very marked in Shakspeare, in Swift, in Sterne, in Cowper. It is traceable in Shenstone, in Johnson, in Southey, and still more in Charles Lamb." A list of names curiously interesting to the psychologist, for, with a solitary exception, each of these men constitutes an illustration of the mental constitution we have analysed; varying, it is true, as to the minor qualities and individual position, but identical as regards the fundamental characteristics. Need we mention details as to Charles Lamb, or Southey, or Cowper, or Sterne, or Swift?

These examples are drawn from too high a type of mind to be at all common; the majority of mankind have no such gifts of intellect and imagination as they. Nevertheless, the law of transmission and development holds good. A merchant under our professional notice affected with hopeless imbecility and general paralysis, the sequel of chronic mania, for years before his mental disorder manifested symptoms of cerebral disease. One of these was, that after smoking a cigar he could not lift his eyelids so as to open his eyes, nor, on some occasions, could he articulate the words he would utter. He took alcoholic drinks in quantity far beyond the powers of resistance of his cerebrum, and fell a victim to their morbific action. Now, this individual has a son and daughter approaching adult life. The former has been subject from childhood, at varying intervals, to paroxysms of extreme terror and distress, arising from no obvious or known cause; very similar to those which attack the oinomaniac, but *as yet* (being but sixteen years of age) without the impulsive desire for stimulants. Previously to the attack there is great irritability and restlessness, with a tendency to sleep, then the outbreak of inexplicable terror commences (usually in the night), continuing for two or three days. When it subsides, he is left weak, ill, and exhausted. The daughter, on the contrary, is passionately fond of every kind of pleasure, as dancing, society, &c.; excels in artistic accomplishments, and is singularly vivacious and animated. Both these children have manifestly derived from their father a cerebral constitution, which will endanger their well-being and happiness as years advance by predisposing to the development of those insane impulses which we have discussed, or to various forms of melancholia.

The deduction from the varied and numerous facts we have placed before the reader is obvious, namely, that both paroxysmal and continuous drunkenness present all the essential characteristics of true mania, but especially the absolute subjection of the will to an impulse or appetite; the subjection of the will and the development of the impulse or appetite being alike *dependent upon, or connected with, morbid conditions of the cerebrum.* What those conditions are, is not so obvious, but whether we look at the nature of the symptoms, the hereditary transmission of the affection, the periodicity of the attacks and the general etiology, we may clearly conclude that the cerebral changes differ little from those occurring in other analogous forms of insanity. These deductions naturally indicate the plan of treatment.

The *treatment of oinomania* will differ much, accordingly as it is paroxysmal or continuous. The great object of treatment will be to restore to the patient the power of self-control, by beneficially modifying those conditions of the cerebrum upon which the development of the appetites or impulses and the subjection of the will depend. The principal means to this end is, undoubtedly, the withdrawal of the individual from the habitual use of the nervine stimuli, the action of which upon the brain is to develop the identical morbid conditions that constitute the disease, or to substitute others for them. But it is precisely in this withdrawal that the great difficulty of treatment, at least of the continuous oinomaniac, consists; for all experience has shown that, if he have freedom of action, no motives whatever are sufficient to restrain him from their use. Curative treatment in establishments devoted to the reception of confirmed drunkards is an idea that has been mooted from time to time. It was discussed in 1834 by the Parliamentary Committee, and, of late years, has had numerous advocates. In our last number we called attention to an attempt now being made at New York to establish such an institution there, to be designated "The United States' Inebriate Asylum." The fundamental principle of management of such institutions must necessarily be the exercise of the same kind of authority over the personal movements of the drunkard as is exercised over the insane in asylums. Sufferers from the disease have also advocated this method of treatment in their own case. In S. T. Coleridge, "the passion for opium had so completely subdued his *will*, that he seemed carried away, without resistance, by an overwhelming flood. The impression was fixed on his mind that he should inevitably die unless he were placed under *constraint*, and that constraint, he thought, could be alone effected in an *asylum!* Dr. Fox, who presided over an establishment of this description in the neighbourhood of Bristol, appeared to Mr. C. the individual to whose subjection he would most like to submit."*

Coleridge was not sent to this asylum, but was placed under medical treatment, and had an attendant whose duty it was to prevent him obtaining that by stealth from which he was openly debarred. Coleridge, however, contrived to evade every precaution, and by various cunning schemes always obtained the desired drug. It is not an usual circumstance for confirmed drunkards thus to *know* at least what is for their good, and to be ready to submit themselves to restraint. Those suffering from the paroxysmal form, Dr. Hutcheson remarks, are "so convinced of the necessity of being controlled, that when the first symptoms of their paroxysm are felt, they voluntarily enter an asylum, and remain till the attack has passed off. These, however, are men of stronger minds, though, with all their strength, incapable of resisting the disease." It is quite certain generally, that in proportion as there is a necessity for curative restraint, in the same proportion will the sufferer's will and intellect be degraded, and no motives will be sufficient to induce a voluntary subjection to control. Who with the slightest practical knowledge of insanity and of the insane can deny that there are many hundreds of persons now under restraint in

* Cottle, *Op. citato.*

asylums, both public and private, who are less dangerous to themselves and to society, more amenable to motives, possessed of more self-control,—more rational, in short, in every respect than the thousands of oinomaniacs who now infest society uncontrolled? wasting their own property and the property of others, ruining their families, displaying without hinderance the "*inhumanitas*," "ferocity," and "moroseness" of the insane drunkard, and transmitting to their wretched off-spring their own morbid cerebral organization, as a Pandora's box from which a host of miserable disorders will inevitably arise. If maniacal irresponsibility be the necessary and proper ground for restraint, then that ground is amply shown and undeniably demonstrated in the natural history and mental pathology of the oinomaniac. As to the general propriety and advantage, therefore, of restraint in these cases, no practical man can doubt.

The *decision* as to the propriety of subjecting any individual drunkard to restraint might be left in the hands of two medical practitioners expressly appointed to that duty, rather than to a jury, provided fixed principles for the guidance of their judgment were laid down. They would have to determine in the first instance the *facts* of the case by personal investigation and inquiry, and from these facts deduce the general conclusion that the sufferer has lost all power of self-control, and is destroying his health to a dangerous extent, utterly neglecting his domestic or social duties, and ruining his patrimony, whether it be in real estate, in personalty, or in the less tangible form of business-connexions. In multitudes of instances, the facts and conclusions would be found to be equally obvious and inevitable.

It is a much more difficult question to determine the *extent* to which seclusion and restraint of the oinomaniac should be carried, for the very obvious reasons that while a very short period of total abstinence from intoxicating drinks often suffices for the restoration of the patient to a rational condition, even when insanity of a decided character has been the result of intemperance, a relapse into drunken habits is almost certain if an early dismissal takes place, which, in fact, it is difficult to avoid; for why should you restrain a person from the exercise of his freedom, it is argued, who is perfectly rational, truly sober, and quite determined never again to yield to temptation? Nor should it be forgotten that popular opinion runs strongly against any detention of the kind whatever, on the ground that it is an invasion of the liberty of the subject, and that a man has a right to get drunk if he likes. In the Report of the Commissioners for 1844, the liberation of patients rendered insane by intemperance is discussed, and the following instance, as one involving great perplexity, is mentioned. At a licensed house in Yorkshire the visiting justices liberated a dangerous lunatic, who had been placed therein at the instance of his wife. The man had been in a state of continued drunkenness for many weeks; he had threatened the life of his wife and child; and two of his brothers had died insane. The compulsory abstinence of the establishment had its proper effect, and when the visiting magistrates saw him, they entered their opinion in the Visitors' book to the effect, that "he appears to be perfectly sane at present, and unless sufficient cause for his further detention be shown to the magistrates assembled in petty sessions, he was to be discharged;" and discharged he was accordingly. The result was, that he again threatened the life of his wife, drove her from his home, and was again placed in the custody of the constables. As precisely the same difficulty would arise in dismissing the ordinary oinomaniac as the drunken madman, we subjoin the results of the experience of the Commissioners in reference to the latter:—

"The difficulty which we have experienced has been to determine for how long a period the patient ought to be detained in confinement after his malady has apparently ceased. We have thought it desirable that he should not be exposed too soon to the temptation of again indulging in strong liquors; it having been almost invariably found that patients of this class, if liberated without having undergone a sufficient probation, are very liable to resort to their former practices, and to relapse. At the same time, we have considered that a lunatic asylum is not a place for the permanent detention of persons who have recovered the use of their reason, and are not obnoxious to the charge of unsoundness of mind, otherwise than on account of their liability afterwards to run into their former excesses when restored to liberty. It has been our practice, in cases of this sort, to liberate the patient after a short confinement, if it be the first attack of insanity from this cause, and if he appear to be aware of his misconduct, and to have a desire to reform his habits. In the event, however, of his being confined a second time owing to the same cause, we have felt that his probation ought to continue for a much longer period; and indeed we have felt great responsibility has rested upon us in such a case, and have at all times very reluctantly, and only after vainly endeavouring to induce the patient's friends to take charge of him, resorted to our power of liberation."—(p. 175.)

The great tendency to relapse is in fact the main difficulty, for it is known that the insane drunkard is specially liable to this. Sir W. Ellis mentioned to the Parliamentary Committee the case of a man dismissed cured from Hanwell. He remained well for twelve months, then began drinking spirits again, and stabbed a policeman, for which he was committed to Newgate. He again recovered, again relapsed, and was re-committed to the same prison for similar misconduct. Mr. William Collins, vice-president of a Scottish Temperance Society, stated to the same Committee, as the result of his experience of drunkards, and as "a well-established physical fact," that the drunkenappetite, when once formed, never becomes completely extinct, but adheres to a man through life.

"If he abstains entirely from spirits, the appetite will not annoy him; its insatiable cravings and the uneasy sensations of the nervous system will cease; but if after ten years' abstinence he take a glass of spirits, his appetite, like tinder, will ignite with the first touch, and flame out again. Hence the danger to which drunkards are exposed, * * * * as we find that at one time or another, when they have been drunkards before, they all fall by the slightest temptation or inducement to taste."

Dr. Hutcheson's experience is very similar to this. He remarks of the chronic form—

"I have seen only one case completely cured, and that after a seclusion of two years' duration. In general it is not cured; and no sooner is the patient liberated than he manifests all the symptoms of the disease. Paradoxical though the statement may appear to be, such individuals are sane only when confined in an asylum."

This practical question is of so great importance that it ought to be placed on a scientific basis by instituting a more careful inquiry into the etiology, pathology, and treatment of oinomania, with a view to what may be termed its *prognosis;* or, in other words, from an examination of the nature, causes, and progress of the disease in each particular case, to deduce safe conclusions as to the amount of self-control that can be ultimately exercised. Although nothing very definite is to be found in books, certain general principles may be deduced from the vast mass of facts recorded. Primarily, and most important of all, arises the question of causation in reference to the condition of the cerebrum. If the brain be permanently defective, then the prognosis is bad, for the organ itself of the will and of the understanding is inherently feeble. Now permanently defective conditions of the cerebrum may be induced by numerous causes. Long continued stimulation by nervine stimuli is one; hence it is that the confirmed drunkard is usually irreclaimable. Injuries to the structure of the brain, whether from mechanical causes, from *coup-de-soleil*, from fever, or from diseases of the encephalon, which induce a constantly recurring morbid condition of the vascular system, as epilepsy, may be placed in the same category. The occurrence of actual insanity, or a known hereditary predisposition thereto, renders the prognosis very doubtful. A natural or inherent condition of the nervous system, such that the appetite for pleasurable feelings is intense, the sufferings from painful sensations great, the foresight defective, and the will feeble, strongly predisposes to relapse. We may here remark incidentally, that persons of this class who have irregularly formed heads and heavy lips, the upper one enlarged, the lower somewhat tumefied and everted, are amongst the most incurable. Oinomania in the parents—one or both—or even what is termed a "moderate" use of spirituous liquors long continued, is of unfavourable omen, for the morbid condition of the cerebrum most assuredly caused by the latter is readily transferred to the children. Where the daughters of a drunkard are nervous and hysterical (as they very often are), and the sons weak, wayward, eccentric, and extravagant, relapses from continuous oinomania are likely to be severe and persistent in any of the family attacked.

*To be continued.* no more.

---

